# Carapanosins D—F from the Seeds of Andiroba (*Carapa guianensis*, Meliaceae) and Their Effects on LPS-Activated NO Production

**DOI:** 10.3390/molecules23071778

**Published:** 2018-07-19

**Authors:** Takanobu Inoue, Shoko Ohmori, Takashi Kikuchi, Takeshi Yamada, Reiko Tanaka

**Affiliations:** Laboratory of Medicinal Chemistry, Osaka University of Pharmaceutical Sciences, 4-20-1 Nasahara, Takatsuki, Osaka 569-1094, Japan; jumbotakoyaki0702@yahoo.co.jp (T.I.); syokentosmar0316@gmail.com (S.O.); t.kikuchi@gly.oups.ac.jp (T.K.); yamada@gly.oups.ac.jp (T.Y.)

**Keywords:** *Carapa guianensis*, Meliaceae, andiroba, seeds, limonoids, *seco*-phragmalin, mexicanolide, carapanosins A–C, NO production

## Abstract

A novel *nor*-phragmalin-type limonoid, named carapanosin D (**1**), and two novel mexicanolide-type limonoids, carapanosins E (2) and F (**3**), were isolated from the seed oil of andiroba *(Carapa guianensis* A_ublet_), a traditional medicine in Brazil and Latin American countries. Their structures were unambiguously determined on the basis of spectroscopic analyses using one-dimensional (1D) and two-dimensional (2D) NMR techniques and High resolution Fast Atom Bombardment Mass Spectrometry (HRFABMS). Compounds **1**–**3** were evaluated for their effects on the production of nitric oxide (NO) in Lipopolysaccharide (LPS)-activated mouse peritoneal macrophages. The NO inhibitory assay suggested that compounds **2** and **3** have high potency as inhibitors of macrophage activation.

## 1. Introduction

Meliaceae plants are a well-known source of structurally diverse limonoids with a wide range of bioactivities, such as antimalarial and antifeedant. Limonoids in the plant kingdom occur mainly in the Meliaceae, Rutaceae, and Simaroubaceae families [1]. Andiroba is one of the Meliaceae plants in the rain forests of South America, and its woody four-cornered nut has four cells, each of which contains two to three seeds with oil-rich kernels. Limonoids, most of which are highly oxidized tetranortriterpenoids, can be classified in terms of *seco* form and cyclization patterns of rings A–D in the triterpene skeleton. Extracts from its flowers, bark, and seeds have been used for centuries by the Amazonian people and exhibit analgesic [2], anti-malarial [3], anti-inflammatory [4], anti-allergic [5], and anti-plasmoidal [6] activities, and also acute and subacute toxicities [7].

Our series of studies on the components of the seed oil of *C. guianensis* revealed the structures of carapanolides A and B [8], guianolide A and B [9], carapanolides C–I [10], carapanolides J–L [11], carapanolides M–S [12], carapanolides T–X [13], and carapanosins A–C [14] in the seed oil of andiroba. Last year, we reported the absolute structure of guianolactones A and B from the seed oil of *C. guianasis* (Meliaceae) [15]. Our recent study of the seed oil of *C. guianasis* revealed the structures of an unusual 19-*nor*-phragmalin-type limonoid, named carapanosin D and two novel mexicanolide-type limonoids, named carapanosins E and F. We herein describe the isolation and structural determination of three new limonoids and the effects of **1**–**3** on the production of nitric oxide (NO) in Lipopolysaccharide LPS-activated mouse peritoneal macrophages. The structures of **1**–**3** were determined on the basis of NMR spectroscopy, including one-dimensional (1D) and two-dimensional (2D) (^1^H, ^1^H-COSY, NOESY, HSQC, HMBC) NMR, and Fast Atom Bombardment (FABMS). 

## 2. Results and Discussion

**Figure 1:**
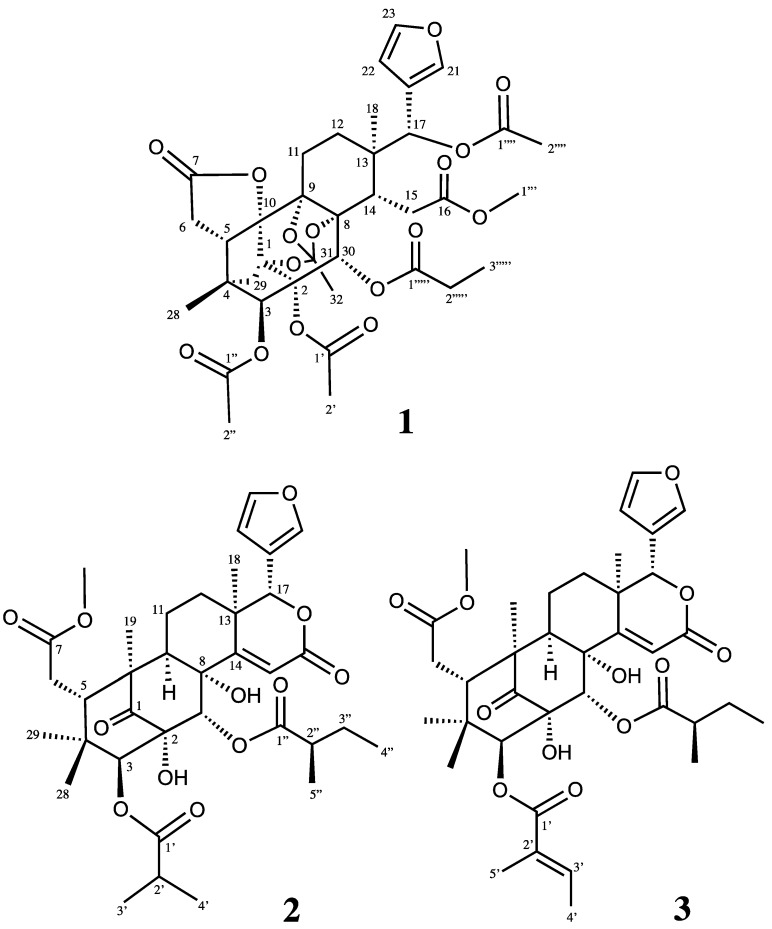
Structures of compounds **1**–**3** from the seeds of *C. guianensis*.

The seeds of *Carapa guianensis* were dissolved in MeOH_,_ and the extract was separated by silica gel column chromatography (CC), medium-pressure liquid chromatography (MPLC), and reverse-phased HPLC to obtain three novel limonoids, **1**, **2**, and **3** (Figure 1). 

**Figure 2:**
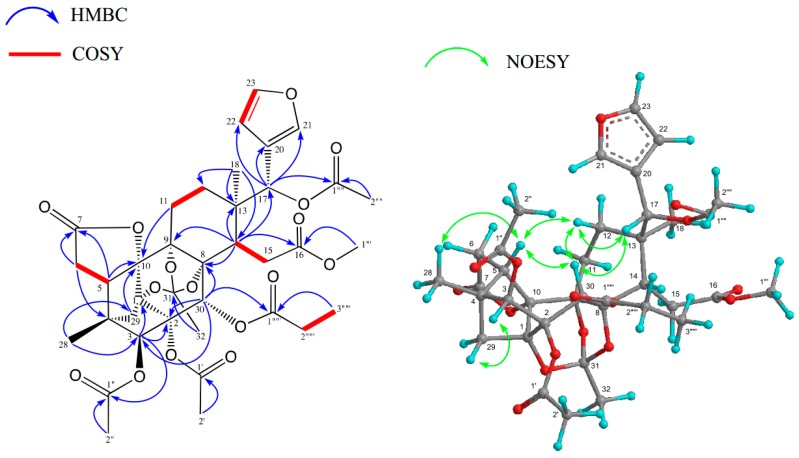
Key HMBC, COSY, and NOESY correlations of Carapanosin D (**1**).

Carapanosin D (**1**) was obtained as a colorless amorphous solid, and possesses a molecular formula of C_37_H_44_O_16_ (*m*/*z* 745.2693 [M + H]^+^, calcd. 745.2707) based on High resolution Fast Atom Bombardment Mass Spectrometry (HRFABMS). In accordance with the molecular formula, 16 degrees of unsaturation came from two carbon—carbon double bonds and six carbonyls; thus, the remaining eight degrees of unsaturation indicated **1** to be octacyclic. The IR absorption implied the presence of several carbonyl groups (*ν*_max_ 1747 and 1633 cm^−1^). The ^1^H and ^13^C-NMR spectra (Table 1) exhibited signals assignable to two tertiary methyls [δ_H_ 0.98, 1.23 (each s)]; three acetyls [δ_H_ 1.96, 2.15, 2.30 (each 3H, s); δ_C_ 21.26, 21.33, 21.6 (each q), 169.3, 169.6, 172.0 (each s)], a propanoyl [δ_H_ 1.20 (3H, t), 2.26 (1H, m), 2.38 (1H, m); δ_C_ 21.3 (q), 28.0 (t), 172.0 (s)], a methyl ester [δ_H_ 3.69 (3H, s); δ_C_ 51.6 (q), 173.9 (s)], an 1,8,9-orthoacetyl group [δ_H_ 1.71 (3H, s), δ_C_ 20.6 (q), 84.0, 84.5, 85.3 (each s), 119.4 (s)] [16], four methylenes, five *sp*^3^ methines including three oxymethine [δ_H_ 5.26 (s), δ_C_ 80.6 (d); 5.68 (s), δ_C_ 69.8 (d); 5.94 (s) δ_C_ 68.7 (d)], six *sp*^3^ quaternary carbons including two oxycarbons [δ_C_ 86.1 (d), 86.4 (s)], a furan [δ_H_ 6.40 (dd), 7.37 (t), 7.68 (brd)], and a lactone [δ_C_ 174.2 (s)]. Analysis of the ^1^H-^1^H COSY spectrum of **1** revealed the partial structure shown in bold face in Figure 2. The HMBC connectivities between H_3_-18 [δ_H_ 1.23 (s)]/C-12, C-13, C-14 and C-17 [δ_C_ 69.8 (d)]; between H-3 [δ_H_ 5.26 (s)]/C-1 [δ_C_ 84.6 (s)], C-2 [δ_C_ 86.1 (s)], C-4, C-5, C-28, C-29, and C-30 [δ_C_ 68.7 (d)]; between H_2_-6 [δ_H_ 2.52 (d)], 2.68 (dd)]/C-4, C-5, C-7 [δ_C_ 174.2 (s)], and C-10 [δ_C_ 86.4 (s)]; between H-14 [δ_H_ 2.36 (dd)]/C-8 [ δ_C_ 85.3 (s)], C-9 [δ_C_ 84.0 (s)], C-12, C-13, C-15, C-16 [δ_C_ 173.9 (s)], and C-30; between H-17 [δ_H_ 5.68 (s) ] /C-12, C-13, C-14, C-20 [δ_C_ 122.5 (s)], C-21 [δ_C_ 142.0 (d)], and C-22 [δ_C_ 109.2 (d)]; between H_2_-29 [δ_H_ 1.7 8 and 1.91 (each d)]/C-1, C-2, C-3, C-4, C-5, and C-10; and between H-30 [δ_H_ 5.94 (s)]/C-2, C-3, C-8, C-9, C-1 4, and C-1′′′′′ [δ_C_ 172.0 (s)] were obtained (Figure 2).

The above NMR data of **1** were similar with those of andirolide O [17], the exclusive difference being lack of C-19 methylene in carapanosin D (**1**), which was confirmed by the HMBC correlations from H_2_-6 and H_2_-29 to the deshielded oxycarbon C-10 [δ_C_ 86.4 (s)], respectively. Therefore **1** would be a 19-*nor* limonoid, and the E ring has a γ-lactone. On the other hand, C-16–C-17 was opened and attached to methylester and acetate, respectively. Thus, the framework of **1** could be a C-19-*nor*, C-16,17-*seco*-phragmalin-1,8,9-orthoacetate. The relative configuration of **1** was determined by the NOESY spectrum, in which significant nuclear Overhauser effect (NOE) were observed between H-3 and H_2_-29; between H-5 and H-12β, H-30, and CH_3_-28; between H-14 and H-11β; between H-17 and H-12β, H-30; between H-30 and H-5, H-12β, H-15β, and H-17; and between CH_3_-18 and H-11, H-12α, and H-22. Therefore, the relative structure of **1** was confirmed as shown in Figure 1. 19-*Nor*-phragmalin was first isolated from *Chukrasia tabularis* by Yin, J-L., et al., who described Tabulvelutin A as a unique 7,10-γ-lactone [18], carapanosin D (**1**) is the second example of 19-*nor*-phragmalin. 

**Figure 3:**
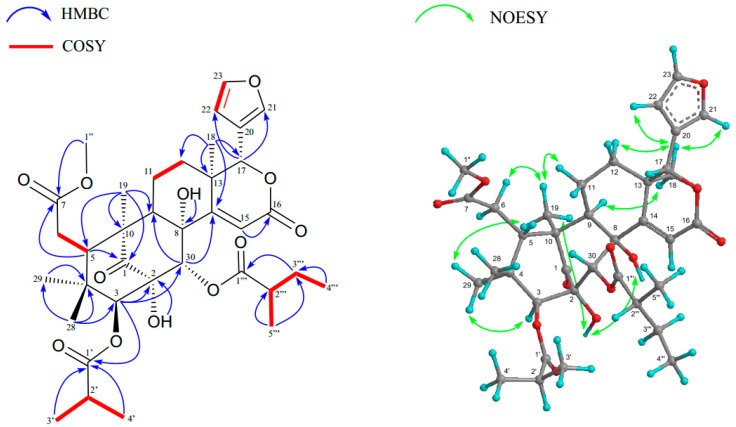
Key HMBC, COSY, and NOESY correlations of Carapanosin E (**2**).

Carapanosin E (**2**) was obtained as a colorless amorphous crystal, and has a molecular formula of C_36_H_48_O_12_ (*m*/*z* 673.3224 [M + H]^+^, calcd. 673.3224) by HRFABMS. The IR absorptions implied the presence of hydroxy, ester, six-membered ring ketone, and αβ-unsaturated δ-lactone at *ν*_max_ 3489, 1727, 1710, and 1670 cm^−1^. ^1^H and ^13^C-NMR spectra (Table 2) revealed the presence of four methyls [δ_H_ 0.83, 0.91, 1.09, 1.28 (each 3H, s)], 2-methylpropanoyl [δ_H_ 1.20 (3H, d), 1.27 (3H, d), 2.86 (1H, sept); δ_C_ 175.5 (s)], 2-methylbutanoyl [δ_H_ 0.87 (3H, t), 1.12 (3H, d), 1.46 m), 1.64 (1H, m), 2.43 (1H, m); δ_C_ 174.4 (s)], a methylester [δ_H_ 3.71 (3H, s); δ_C_ 52.3 (q), 173.8 (s)], an αβ-unsaturated δ-lactone [δ_H_ 6.34 (1H, s), δ_C_ 115.5 (d), 164.9 (s), 165.8 (s)], a six-membered ring ketone [δ_C_ 204.1 (s)], two tertiary hydroxyls that disappear by heavy water processing [δ_H_ 2.84, 4.08 (each 1H, s)], and a β-substituted furan ring [δ_H_ 6.47 (dd), 7.44 (t), 7.45 (d)], therefore, **2** could be suggested as a mexicanolide-type limonoid. The HMBC connectivities between H_3_-18 [δ_H_ 1.28 (s)]/C-12, C-13, C-14 [δ_C_ 165.8 (s)], and C-17 [δ_C_ 78.9 (d)]; H_3_-19 [δ_H_ 1.09 (s)]/C-1 [δ_C_ 204.1 (s)], C-5, C-9, and C-10; H-3 [δ_H_ 5.15 (s)]/C-1, C-2 [δ_C_ 86.3 (s)], C-4, C-5, C-28, C-29, and C-30 [δ_C_ 73.9 (d)]; H-15 [δ_H_ 6.34 (s)]/C-8 [δ_C_ 80.6 (s)], C-13, C-14, and C-16 [δ_C_ 164.9 (s)]; H-17 [δ_H_ 5.44 (s)]/C-12, C-13, C-16, C-20 [δ_C_ 120.3 (s)], C-21 [δ_C_ 141.6 (d)], and C-22 [δ_C_ 110.5 (d)]; H-30 [δ_H_ 6.51 (s)]/C-1, C-2, C-3 [δ_C_ 79.7 (s)], C-8, C-9, and C-14; 2-O*H* (δ_H_ 4.08 (s)/C-1, C-2, and C-30; 8-O*H* (δ_H_ 2.84 (s)/C-8, C-9, C-14, and C-30 were observed. In the ^1^H-^1^H COSY spectrum, five distinct spin sets of H-5–H-6; H-9–H_2_-11–H_2_-12; H-22–H-23; H_3_-3′–H-2′–H_3_-4′; and H_3_′′′′-5–H-2′′′′–H_2_-3′′′′–H_3_-4′′′′ were observed (Figure 3). These results estimate the plain structure of **2** as shown in Figure 3. The relative configuration of **2** was mainly established by a NOESY experiment. It has strong cross-peaks of H_3_-18/H-9α, H-12α, H-15, H-21, and H-23; H_3_-19/H-6α, H-9α, H-11α, H_3_-29, and 2-OH; 8-OH/2- OH; H-3/H-6α, and H_3_-29, therefore, the relative structure was established as shown in Figure 1. The configuration of 2-methylbutanoyl group at C-30 was deduced to be *R* because the chemical shift value of Me-5’” [δ_H_ 1.12 (d, *J* = 7.2 Hz); δ_C_ 16.7 (q)] were in accordance with those of carapanolide F [δ_H_ 1.02 (d, *J* = 7.2 Hz); δ_C_ 16.0 (q)] [10], which was determined as *R* by a single-crystal X-ray diffraction analysis.

Carapanosin F (**3**) has the molecular formula C_37_H_48_O_12_ (*m*/*z* 673.3224 [M + H]^+^, calcd. 673.3224) as determined by HRFABMS. The UV, IR spectra showed αβ-unsaturated δ-lactone and hydroxyl, ester, and a six-membered ring ketone [UV λ_max_ (CH_3_CN) nm (log ε): 232 (3.82); IR ν_max_ cm^−1^ (KBr): 3462, 1727, 1707]. NMR data were very similar to those of **2** except for a tigroyl group [δ_H_ 1.91 (s), 1.92 (d), 6.88 (m); δ_C_ 12.4 (q), 14.7 (q), 128.8 (s), 138.2 (d)] at C-3. NOESY spectrum revealed the relative stereochemistry of **3** to have the same conformation as **2**.

**Figure 4:**
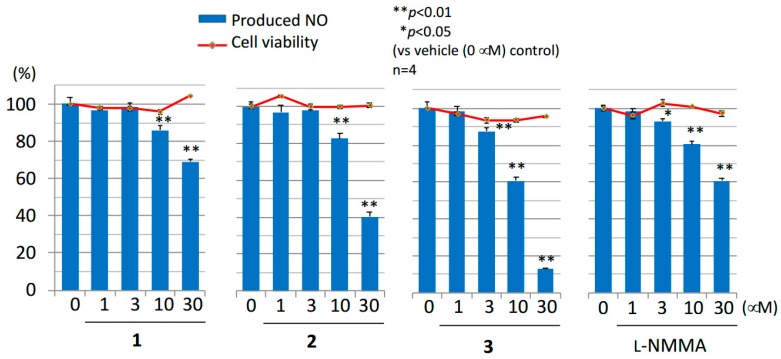
Inhibitory activities on nitic oxide (NO) production and cytotoxicities of Compounds **1**–**3** and N^G^-monomethyl-L-arginine acetate (l-NMMA). Each value represents the mean and the standard error (S.E.) of four determinations. Significant differences from the vehicle control (0 μM) group shown as: * *p* < 0.05 and ** *p* < 0.01 in the NO inhibitory assay.

Physiological nitric oxide (NO) plays important roles in blood pressure regulation and blood flow distribution. However, its overexpression may cause multiple organ dysfunction, tissue injury, and systemic inflammatory responses in sepsis, such as hypotension, vascular hyporeactivity, and cardiodepression [19]. In this study, three limonoids and N^G^-monomethyl-L-arginine acetate (l-NMMA), which is an inducible nitric oxide synthase (iNOS) inhibitor, were assayed for their inhibitory effects on NO production in LPS stimulated RAW 264.7 cells. Cytotoxicities of limonoids tested were evaluated by the [3-(4,5-dimethylthial-2-yl)-2,5-diphenyltetrazalium bromide] (MTT) assay for determination of safe concentrations. Mexicanolide-type limonoids **2** and **3** exhibited stronger inhibitory activities (IC_50_ of NO produced **2**: 23.9 μM; **3**: 11.8 μM) than the positive control, l-NMMA (IC_50_ of NO produced 47.6 μM) without cytotoxicities (Figure 4). These results demonstrated that compounds **2** and **3** have potency as inhibitors of NO production. However, the effect of compounds **1** and **2** were inferior to gedunin type limonoids such as gedunin (IC_50_ of NO produced **2**: 4.6 μM), 6α-acetoxygedunin (IC_50_ 7.9 μM), 7-deacetoxy-7-hydroxygedunin (IC_50_ 8.7 μM), and 6α-acetoxy-7α-deacetoxy-7α-hydroxygedunin (IC_50_ 9.4 μM) from the flower oil of *C. guianensis* [20].

## 3. Experimental Section

### 3.1. General Procedures

Melting points were determined on a Yanagimoto micro-melting point apparatus (YANAKO Measuring Instrument Trading Corporation, Kyoto, Japan) and were uncorrected. Optical rotations were measured using a JASCO DIP-1000 digital polarimeter (JASCO Corporation, Tokyo, Japan). IR spectra were recorded using a PerkinElmer 1720X FTIR spectrophotometer (PerkinElmer Japan Co. Ltd., Yokohama, Japan). All NMR experiments were measured with a Varian INOVA 600 spectrometer (Varian Medical Systems, Tokyo, Japan) with standard pulse sequences, operating at 600 and 150 MHz. CDCl_3_ was used as the solvent and Tetramethylsilane (TMS) as the internal standard. FABMS were recorded on a JEOL-7000 mass spectrometer (70 eV) (JEOL Ltd., Tokyo, Japan). Column chromatography (CC) was carried out on silica gel 60 (70–230 mesh) (Merck Chemicals B.V., Tokyo, Japan) and MPLC was carried out with silica gel (230–400 mesh) (Merck Chemicals B.V., Tokyo, Japan). HPLC was completed using a JASCO PU-1586 instrument (JASCO Corporation, Tokyo, Japan) equipped with a differential refractometer (RI 1531). Fractions obtained from column chromatography were monitored by thin-layer chromatography (TLC) (silica gel 60 F_254_) (Merck Chemicals B.V., Tokyo, Japan). 

### 3.2. Plant Material

The seed oil (2.03 kg) of Andiroba (*Carapa guianensis* A_ublet_, Meliaceae) was collected in the Amazon, Brazil, in March, 2013. It was kindly provided by Mr. Akira Yoshino (who is a representative person of the “NGO Green Heart love Amazon project”). A voucher specimen (CGS-01-2) was deposited in the Herbarium of the Laboratory of Medicinal Chemistry, Osaka University of Pharmaceutical Sciences.

### 3.3. Isolation of Compounds ***1**–**3***

The seed oil of Andiroba (*Carapa guianensis* Aublet, Meliaceae) (2.03 kg) was dissolved in CHCl_3_, and the CHCl_3_ solution was subjected to CC (silica gel 14 kg), and affording 7 fractions: Fractions A (Fr. No. 1–76, 900 g), B (Fr. No. 77–110, 12.0 g), C (Fr. No. 111–125, 21.0 g), D (Fr. No. 126–155, 10.9 g), E (Fr. No. 156–170, 1.4 g), F (Fr. No. 171–180, 2.4 g), G (Fr. No. 181–195, 2.9 g), and H (Fr. No. 196–208, 0.7 g) [15]. Fraction D was rechromatographed over a silica gel open-column (230–400 mesh, 200 g) eluted with *n*-hexane–AcOEt (1:1) to give eight fractions: D(1) (Fr. No. 1–35, 4.52 g), D(2) (Fr. No. 36–49, 1.81 g), D(3) (Fr. No. 50–88, 1.40 g), D(4) (Fr. No. 89–115, 0.93 g), D(5) (Fr. No. 116–130, 0.60 g), D(6) (Fr. No. 131–140, 0.52 g), D(7) (Fr. No. 141–205, 0.47 g), and D(8) (Fr. No. 206–215, 0.24 g). Fraction D(4) was subjected to a silica gel open-column (230–400 mesh, 100 g) eluted with *n*-hexane–EtOAc (3:1) to give an amorphous solid (34.1 mg) that was purified by HPLC (ODS, 75% MeOH) to give compounds **2** (6.2 mg, Retention time: 53.3 min.) and **3** (1.79 mg, Retention time: 52.5 min.). Fraction D(5) was subjected to a silica gel open-column (230–400 mesh, 60 g) eluted with *n*-hexane–EtOAc (3:1) to give an amorphous solid (24.0 mg) that was purified by HPLC (ODS, 75% MeOH) to give compound **1** (4.5 mg, Retention time: 42.8 min.).

### 3.4. Analytical Data

Carapanosin D (**1**): Colorless amorphous solid; ${\left[\alpha \right]}_{D}^{20}$ −9.5° (*c* = 0.1, EtOH); HRFABMS *m*/*z*: calcd. for C_37_H_44_O_16_, [M + H]^+^: 745.2693; found 745.2707; IR (KBr) ν_max_ cm^−1^; 2975, 1747(O–C=O), 1633; for ^1^H and ^13^C-NMR spectroscopic data, see Table 1; FABMS *m*/*z* (rel. int.): 745 (100), [M + H]^+^, 685 [M + H − HOAc]^+^ (72), 449 (33).

Carapanosin E (**2**): Colorless amorphous solid; m.p. 96–98 °C; ${\left[\alpha \right]}_{D}^{26}$ −25.8° (*c* 0.1, CHCl_3_); HRFABMS: *m*/*z* calcd for C_36_H_49_O_12_ [M + H]^+^: 673.3224; found 673.3224; UV λ_max_ (CH_3_CN) nm (log ε): 219 (3.76); IR (KBr) ν_max_ cm^−1^: 3489 (OH), 2974, 1727 (O–C=O), 1710 (six membered ring ketone), 1670 (αβ-unsaturated δ-lactone and 1461; for ^1^H and ^13^C-NMR spectroscopic data, see Table 2; FABMS *m*/*z* (rel. int.): 673 (27) [M + H]^+^, 57 (100). 

Carapanosin F (**3**): Colorless amorphous solid; m.p. 83–85 °C; ${\left[\alpha \right]}_{D}^{26}$ +16.6° (*c* 0.1, CHCl_3_); HRFABMS *m*/*z* calcd. for C_37_H_49_O_12_ [M + H]^+^: 685.3224; found 685.3224; UV λ_max_ (CH_3_CN) nm (log ε): 232 (3.82), IR (KBr) ν_max_ cm^−1^: 3462 (OH), 2970, 1727 (O–C=O), 1707 (six membered ring ketone), 1670 (αβ-unsaturated δ-lactone), 1549, and 1461; for ^1^H and ^13^C-NMR spectroscopic data, see Table 2; FABMS *m*/*z* (rel. int.): 685 (11) [M + H]^+^, 83 (100). 

### 3.5. Cell Cultures

RAW264.7 cells (mouse macrophages) purchased from DS Pharma Biomedical Co., Ltd. (Osaka, Japan) were incubated in Dulbecco’s Modified Eagle Medium (DMEM) containing 10% Fetal Bovine Serum (FBS) and antibiotics (100 units/mL penicillin and 100 μg/mL streptomycin) in a 5% CO_2_ humidified incubator at 37 °C. 

### 3.6. Determination of RAW264.7 Cell Proliferation

RAW264.7 cell proliferation was examined as described previously [13].

### 3.7. Inhibitory Assay of NO Production

An inhibitory assay of nitric oxide production was performed as describe previously [13].

## 4. Conclusions

A novel *nor*-phragmalin-type limonoid, named carapanosin D (**1**), and two new mexicanolide-type limonoids, named carapanosins E and F (**2**, **3**) were isolated from the seeds of *Carapa guianensis* (andiroba). Their structures were elucidated by extensive spectroscopic techniques. Carapanosin D (**1**) is the second example of 19-*nor*-phragmalin. Compounds **1**–**3** showed non-toxicities at 0–30 μM. Of these, compounds **2** and **3** showed superior inhibitory activities (IC_50_ of NO produced **2**: 23.9 μM; **3**: 11.8 μM) compared to the positive control, l-NMMA (IC_50_ of NO produced 47.6 μM). These results suggest that compounds **2** and **3** have high potency as inhibitors of macrophage activation.

## Figures and Tables

**Table 1:** ^1^H- (600 MHz) and ^13^C- (150 MHz) NMR spectroscopic data of compound **1**.

Position		1			Position		1		
		^1^H ^a^ (*J*, Hz)	^13^C ^b^	HMBC			^1^H ^a^ (*J*, Hz)	^13^C ^b^	HMBC
1			84.6 (s)		18		1.23	44.8 (q)	12, 13, 14, 17
2			86.1 (s)		20			122.5 (s)	
3		5.26 s	80.6 (s)	4, 5, 28, 30, 1″	21		7.68 brd (0.9)	142.0 (s)	17, 20, 22
4			44.6 (s)		22		6.40 dd (0.6, 1.7)	109.2 (d)	20, 23
5		2.82 d (10.1)	38.2 (d)	1, 3, 4, 6, 7, 10, 29	23		7.37 t (1.7)	143.2 (d)	20, 22
6	α	2.52 d (19.3)	30.0 (t)	4, 5, 7, 10	28		0.98	14.5 (q)	
	β	2.68 dd (10.1, 19.3)			29	*pro-R*	1.91	37.8 (t)	1, 2, 4, 5, 28
7			174.2 (s)		29	*pro-S*	1.78		
8			85.3 (s)		30		5.94 s	68.7 (d)	1, 2, 3, 8, 9, 14
9			84.0 (s)		31			119.4 (q)	
10			86.4 (s)		32		1.71 s	20.6 (q)	
11	α	1.82 m	24.7 (t)	8, 9, 10, 12, 13	1′			170.1 (s)	
	β	1.84 m			2′		2.15 s	21.6 (q)	1′
12	α	1.05 ddd (1.4, 7.1, 14.4)	31.5 (t)	9, 11, 13, 14, 17	1″			169.6 (s)	
	β	1.11 (2.9, 4.7, 14.4)			2″		2.30	21.33 (q)	1″
13			39.1 (s)		1″′		3.69 s	51.6 (q)	16
14		2.36 dd (7.6, 16.5)	47.6 (d)	8, 13, 15, 16, 17, 18, 30	1″″			169.3 (s)	
15	α	2.84 dd (4.1, 16.5)	30.4 (t)	8, 13, 14, 16	2″″		1.96 s	21.26 (q)	1″″
	β	2.20 m			1″″′			172.0 (s)	
16			173.9 (s)		2″″′		2.26, 2.38	28.0 (t)	1″″′
17		5.68 s	69.8 (d)	12, 13, 14, 20, 21, 22, 1″″	3″″′		1.20 t (7.3)	21.3 (q)	1″″′

^a^ Measured at 600 MHz in CDCl_3_. ^b^ Measured at 150 MHz in CDCl_3_. Assignment are based on HMBC spectrum.

**Table 2:** ^1^H and ^13^C-NMR spectroscopic data of compounds **2** and **3** (600 MHz, CDCl_3_, 150 MHz).

Position		2		3	
		^1^H ^a^ (*J,* Hz)	^13^C ^b^	^1^H ^a^ (*J,* Hz)	^13^C ^b^
1			204.1 (s)		204.0 (s)
2			86.3 (s)		86.2 (s)
3		5.15 s	79.7 (d)	5.14 s	80.4 (d)
4			43.4 (s)		43.6 (s)
5		2.62 dd (6.7, 1.5)	38.6 (d)	2.68 t (1.0)	38.9 (d)
6	α	2.45 dd (18.2, 1.5)	32.9 (t)	2.39 t (1.0)	32.9 (t)
	β	2.34 dd (18.2, 6.7)		2.46 t (1.0)	
7			173.8 (s)		173.9 (s)
8			80.6 (s)		80.4 (s)
9		2.47 dd (12.9, 6.2)	65.7 (d)	2.45 m	65.4 (d)
10			55.1 (s)		55.7 (s)
11	α	1.71 m	19.9 (t)	1.72 m	20.0 (t)
	β	1.50 m		1.48 m	
12	α	1.56 m	30.1 (t)	1.54 m	30.2 (t)
	β	1.76 m		1.77 m	
13			39.3 (s)		39.3 (s)
14			165.8 (s)		166.0 (s)
15		6.34 s	115.5 (d)	6.22 s	115.4 (d)
16			164.9 (s)		164.8 (s)
17		5.44 s	78.9 (d)	5.43 s	78.9 (d)
18		1.28 s	21.2 (q)	1.27 s	21.3 (q)
19		1.09 s	18.8 (q)	1.09 s	18.8 (q)
20			120.3 (s)		120.3 (s)
21		7.44 t (1.8)	141.6 (d)	7.45 dd (0.1, 0.2)	141.7 (d)
22		6.47 dd (1.8, 0.9)	110.5 (d)	6.47 dd (0.1)	110.5 (d)
23		7.45 d (0.9)	143.0 (d)	7.44 t (0.2)	143.0 (d)
28		0.83 s	25.0 (q)	0.92 s	21.3 (q)
29		0.91 s	21.4 (q)	0.86 s	25.5 (q)
30		6.51 s	73.9 (d)	6.36 s	74.4 (d)
1’			175.5 (s)		166.2 (s)
2’		2.86 sept (7.1)	34.3 (d)		128.8 (s)
3’		1.20 d (7.1)	18.1 (q)	6.88 q (7.1)	138.2 (d)
4’		1.27 d (7.1)	19.8 (q)	1.91 d (7.1)	12.4 (q)
5’				1.92 s	14.7 (q)
1”		3.71 s	52.3 (q)	3.72 s	52.3 (q)
1”’			174.4 (s)		174.1 (s)
2”’		2.43 m	40.8 (d)	2.39 m	40.7 (d)
3”’	A	1.46 m	26.5 (t)	1.43 dq (1.3, 1.2)	26.5 (t)
	B	1.64 m		1.60 dq (1.3, 1.2)	
4”’		0.87 t (7.2)	11.4 (q)	0.84 t (7.1)	11.3 (q)
5”’		1.12 d (7.2)	16.7 (q)	1.09 d (7.1)	16.7 (q)
2-OH		4.08 s		4.08 s	
8-OH		2.84 s		2.83 s	

^a^ Measured at 600 MHz in CDCl_3_. ^b^ Measured at 150 MHz in CDCl_3_. Assignments are based on HMBC spectrum.
